# Glycosylation Changes in Prostate Cancer Progression

**DOI:** 10.3389/fonc.2021.809170

**Published:** 2021-12-24

**Authors:** William Butler, Jiaoti Huang

**Affiliations:** Department of Pathology, Duke University School of Medicine, Durham, NC, United States

**Keywords:** prostate cancer, glycobiology, biomarker discovery, cancer biology, omics

## Abstract

Prostate Cancer (PCa) is the most commonly diagnosed malignancy and second leading cause of cancer-related mortality in men. With the use of next generation sequencing and proteomic platforms, new biomarkers are constantly being developed to both improve diagnostic sensitivity and specificity and help stratify patients into different risk groups for optimal management. In recent years, it has become well accepted that altered glycosylation is a hallmark of cancer progression and that the glycan structures resulting from these mechanisms show tremendous promise as both diagnostic and prognostic biomarkers. In PCa, a wide range of structural alterations to glycans have been reported such as variations in sialylation and fucosylation, changes in branching, altered levels of Lewis and sialyl Lewis antigens, as well as the emergence of high mannose “cryptic” structures, which may be immunogenic and therapeutically relevant. Furthermore, aberrant expression of galectins, glycolipids, and proteoglycans have also been reported and associated with PCa cell survival and metastasis. In this review, we discuss the findings from various studies that have explored altered *N-* and *O-*linked glycosylation in PCa tissue and body fluids. We further discuss changes in *O*-GlcNAcylation as well as altered expression of galectins and glycoconjugates and their effects on PCa progression. Finally, we emphasize the clinical utility and potential impact of exploiting glycans as both biomarkers and therapeutic targets to improve our ability to diagnose clinically relevant tumors as well as expand treatment options for patients with advanced disease.

## Introduction

Prostate Cancer (PCa) is the most common non-cutaneous malignancy and second leading cause of cancer-related mortality in men over the age of 50 (1). Although most men are diagnosed with low-grade or indolent tumors that are unlikely to metastasize and lead to death, a significant subset of patients develops recurrence of their tumor following local intervention or, more rarely, are diagnosed with distant metastases at clinical presentation (2, 3). Since the vast majority of PCa cells express high levels of androgen receptor (AR) (4), hormonal therapy (i.e. androgen ablation or AR inhibition) remains the mainstay of treatment for men diagnosed with recurrent or advanced disease (2). Unfortunately, the tumor eventually becomes resistant to treatment in all cases which is classified as castration-resistant prostate cancer (CRPC) (5). CRPC is associated with a poor prognosis as the tumor cells are more proliferative and display a higher capacity for metastasis (5). Furthermore, treatment for CRPC remains very limited due to an overall lack of mechanistic understanding which in part is due to limited tissue resources as such tumors rarely undergo biopsy or resection.

Many studies have been performed to molecularly profile prostatic tumors to address two clinical situations: 1) Stratify patients with primary PCa into different risk groups for optimal management and 2) Elucidate the molecular features of CRPC to inform novel therapeutic strategies. In recent years, several studies with large patient cohorts (6–9) have been done to survey the molecular landscape in these contexts. Most of these involve the use of RNA or single-cell sequencing as well as proteomic analysis to study differential gene and protein expression amongst different disease stages. Whilst these studies provide powerful information on how the molecular landscape changes during PCa progression, it is becoming increasingly recognized that the glycome is also a significant source of biomarkers and therapeutic targets (10) that has remained relatively unexplored in many cancer settings including PCa. Aberrant glycosylation is a well-known hallmark of cancer and is known to have a significant effect on protein function and cell survival (10). Furthermore, the prostate is a major secretor of glycoproteins and significant changes in the structures of both cell surface and secreted glycans have been reported by several groups (11). In this review, we summarize the wide range of glycosylation changes observed as PCa progresses including changes in *N*- and *O*-linked glycans, *O*-GlcNacylation, as well as altered expression of galectins and glycoconjugates such as heparan and chondroitin sulfate proteoglycans (HSPGs and CSPGs) and glycolipids. Furthermore, we highlight new technological advances in studying glycobiology in human tissue and how this has been applied to PCa. By providing both historical context and modern perspective, this review hopes to emphasize how we may exploit glycans as biomarkers and therapeutic targets to advance the field of PCa biology.

## *N*-linked Glycosylation in Prostate Cancer

**Figure 1:**
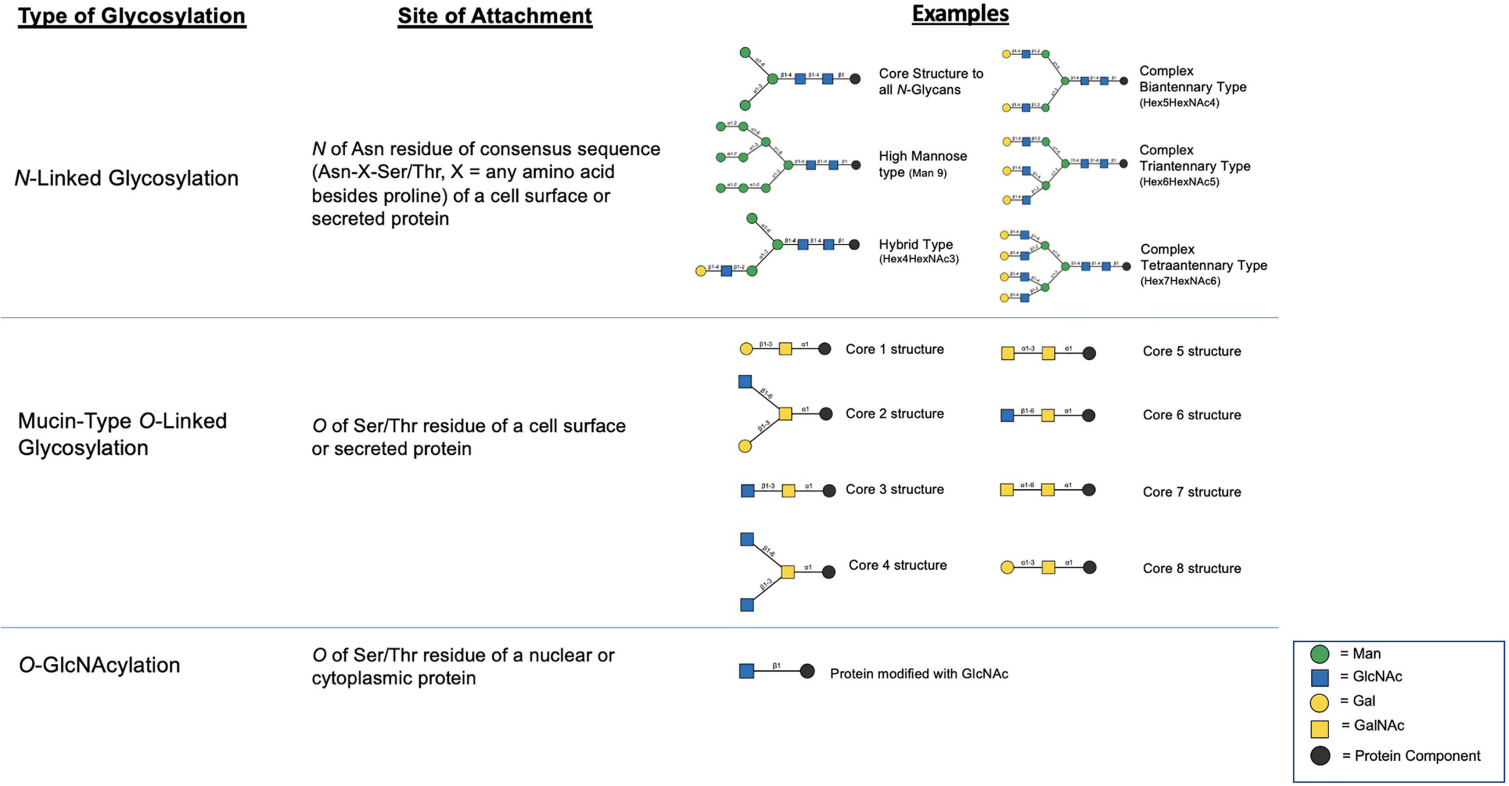
Summary of major classes of glycosylation including examples of structural products resulting from the various mechanisms (12).

*N*-linked glycosylation is a process by which a pre-assembled group of 14 saccharides are co-translationally added to an asparagine (Asn) residue of a protein at a defined motif (Asn-X-Ser/Thr, where X is any amino acid besides proline) and then post-translationally modified in the Golgi apparatus by several glycosyltransferases and glycosidases which can result in a wide variety of unique structures (**Figure 1**) (13). Given the large number of glycosyltransferases/glycosidases that catalyze the addition or release of specific saccharide linkages (13), there is a large degree of structural heterogeneity that may be present in a given cell type. However, in most settings, the abundance of specific *N*-glycan structures is highly consistent in a given tissue type (13) and significant changes in the abundance of specific *N*-glycans is known to occur in cancer (10). This likely offers a survival benefit to cells and is thought to be due to mutations, changes in expression, epigenetic regulation, or posttranslational modifications to involved enzymes in *N*-glycan biosynthetic pathways (10, 11). In this section, we summarize the major reported changes in *N*-linked glycosylation derived from both broad profiling studies as well as targeted studies focusing on changes in glycosylation of select, common PCa biomarkers (PSA, PAP, PSMA). Furthermore, we highlight recent technological advances that have been made in studying the *N*-glycome and potential applications in the area of molecular histology.

### Broad Changes in *N*-Glycosylation Associated With PCa

Gleason scoring is currently the most commonly used metric for predicting prognosis in men newly diagnosed with PCa (14). With this system, a pathologist renders a score (1-5) based on the most prevalent growth pattern and the second most prevalent growth pattern (1 = well differentiated, 5 = poorly differentiated). The two scores are then added together with the first score listed being the most prevalent pattern (i.e. a score of 3 + 4 = 7 indicates that grade 3 pattern is more prevalent than grade 4). Men with higher Gleason scores (GS ≥ 8) are generally assumed to have a higher chance of disease recurrence followed by metastasis compared to men with lower scores (14). Despite the utility of Gleason scores in stratifying men into different risk groups, the disease course for a specific patient, particularly one with an intermediate score (i.e. GS = 3 + 4 or 4 + 3) can be unpredictable (14, 15). Therefore, many research efforts have been made to discover new biomarkers that can provide prognostic information independent of Gleason score.

Many studies characterized how the *N*-glycosylation profile changes correlate with PCa biology to discover new biomarkers often using the Gleason score system as a metric for disease aggressiveness. Many of these earlier studies, although insightful, did not characterize the glycan structures themselves but instead looked at changes in the levels of glycoproteins. For example, one of the earlier studies utilized OCT-embedded prostate tissues from patients with either non-aggressive PCa (GS = 6 or GS = 7 with no evidence of recurrence in 15 years) or aggressive PCa (GS = 7 with disease recurrence within 6 years or GS ≥ 8) and selected for *N*-glycans using a solid-phase extraction method followed by mass spectrometric (MS) analysis (16). From 350 formerly *N*-linked glycopeptides, 17 appeared to be differentially expressed between aggressive and non-aggressive PCa. In particular, an *N*-glycosite signature associated with aggressive PCa emerged from this approach where the expression of COMP and periostin was found to be increased and the expression of VAP-1 was decreased (16). Expanding on this work, a follow-up study was published by the same group with a larger cohort and wider variety of patient samples (17). Here, *N*-linked glycopeptides were isolated from tissue representing normal prostate (n = 10), non-aggressive PCa (n = 24), aggressive PCa (n = 16), and metastatic PCa (n = 25) and analyzed using SWATH mass spectrometry. In this study, 1430 *N*-glycosites were identified per sample and 220 showed significant quantitative changes associated with PCa progression and metastasis. Two glycoproteins in particular, N-acylethanolamine acid amidase and protein tyrosine kinase 7, were significantly associated with aggressive PCa and further validation in patient tissue showed the two markers to be highly predictive of advanced disease (17).

High throughput profiling of *N*-glycans on human PCa tissue has historically only been performed on small sample sizes, perhaps due to technological limitations and limited tissue resources. In 2014, Powers et al. utilized MALDI-imaging mass spectrometry (MALDI-IMS) to profile glycans directly on human PCa tissue (18). This powerful technology allows detected glycans to be spatially mapped to specific tissue regions. Here, a human PCa tissue block with both tumor and non-tumor regions was analyzed and it was shown that high mannose glycans (Man5-Man9) were particularly evident in the tumor regions compared to adjacent benign and stroma while multiple biantennary non-sialylated glycans were detected primarily in non-tumor regions (18). A recent study utilized MALDI-IMS to profile a tissue microarray containing prostate tumor tissue (n = 108) and benign tissue (n = 30) from 138 patients (19). Here, high mannose glycans were found to be abundant in tumor regions as well as increased tri- and tetraantennary glycans that increased proportionally with tumor grade (19). Furthermore, the triantennary glycan at 2,320 m/z was found to be highly abundant in patients with biochemical recurrence and was correlated with decreased survival by Kaplan-Meier analysis (19). Although not global approaches, several historical studies have utilized human PCa tissue to survey presence or absence of Lewis antigens which are highly associated with malignant transformation (20–24). These studies indicate reduction of the lewis A/B family of antigens in prostatic adenocarcinoma relative to benign prostate but the presence of sialyl lewis X and lewis Y in metastatic cancer. Furthermore, sialyl lewis X was shown to have a strong association with poor prognosis in men who have undergone hormonal therapy (24).

Although tissue studies remain limited, there is an abundance of studies that have profiled *N*-glycan structures from PCa patient serum. In a study comparing the serum from men with benign prostate hyperplasia (BPH) (n = 13) to men with PCa (n = 34), it was found that core-fucosylated biantennary glycans and α2-3 sialic acids were significantly increased in PCa relative to BPH (25). On the other hand, triantennary trigalactosylated glycans and tetraantennary tetrasialylated glycans with outer arm fucose showed a significant decrease compared to BPH (25). An earlier study attempted to profile the glycome in men receiving androgen deprivation therapy (ADT) using MALDI-MS of permethylated glycans released by PNGase F cleavage (26). Compared to healthy men (n = 10), men receiving ADT (n = 24) showed an overall decrease in smaller *N*-glycans and a significant increase in multiantennary glycans. Furthermore, overall fucosylation was increased in the ADT group (26). Interestingly, FUT8, the enzyme solely responsible for mammalian *N*-acetylglucosamine I core fucosylation, was found by another group to result in androgen-independent cell survival when overexpressed, potentially linking *N*-glycosylation to the CRPC phenotype (27). This was found to be associated with up-regulation of EGFR and downstream signaling, suggesting that core fucosylation may result in a “switch” from AR-driven signaling to EGFR-driven signaling in hormone-depleted conditions, allowing increased cell survival.

In a recent study, whole-serum glycome profiling was carried out on 117 PCa patients’ serum using ultra-performance liquid chromatography (UPLC) to separate *N*-glycans released from serum glycoproteins (28). Results of this study indicated an increase in hybrid, high mannose, and biantennary digalactosylated monosialylated glycans (M5AG1S1, M8, and A2G2S1) with a decrease in triantennary trigalactosylated trisialylated glycans with and without core fucose (A3G3S3 and FA3G3S3) with PCa progression from indolent through significant/aggressive disease (28). Although insightful, none of the patients in the study received hormonal therapy and were categorized using Epstein’s criteria post-prostatectomy. Another recent study examined serum *N*-glycans by glycoblotting from healthy volunteers (n = 80), BPH (n= 286), early-stage PCa (n = 258), PCa being treated with ADT (n = 46), and CRPC (n = 68) (29). Similar to the results obtained by Kyselova et al. (26), this study found elevated levels of tri and tetra antennary glycans in CRPC serum and found this to also be predictive of developing CRPC in the ADT group (29). Collectively, the results of these studies suggest that increased branching of *N*-glycans may be an important mechanism for CRPC and is deserving of further study. However, it is important to note that the serum *N*-glycome does not necessarily indicate the glycans are derived directly from the prostate as this could be due to an effect on other tissue-types as a result of hormonal therapy. Further studies will be needed to elucidate whether these *N*-glycans are indeed prostate-derived.

**Figure 2:**
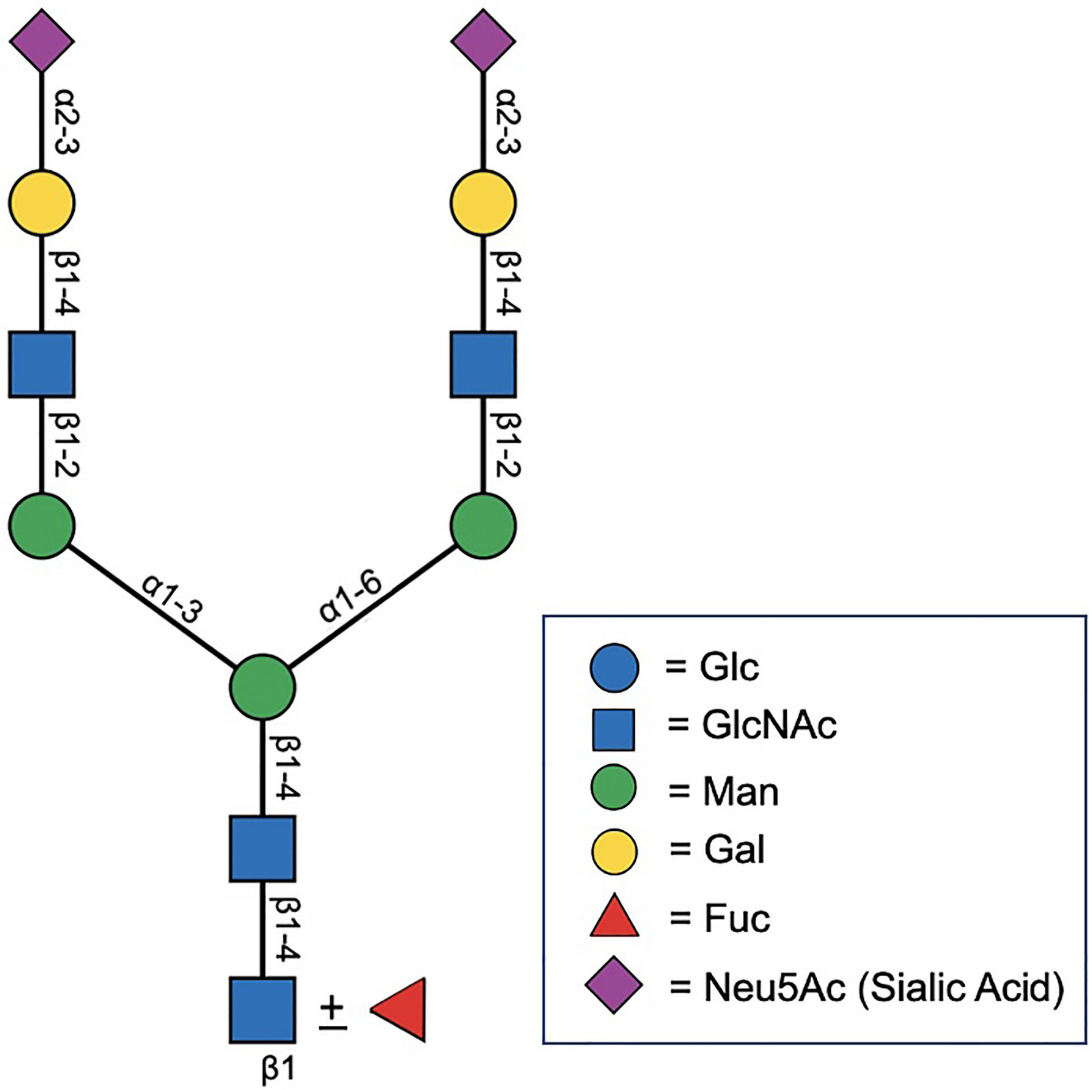
Biantenarry *N*-glycan consisting of two terminally sialyted lactosamine groups with and without core fucose, representative of the most common glycoform found on PSA (12).

Expressed prostatic secretions (EPS) and urine are also important sources of glycoconjugates (11). In addition to several proteomic studies that have shown an abundance of *N*- and *O*-linked glycoproteins in EPS (30–32), direct *N*-glycan analysis was performed on EPS-urine derived from men with negative biopsy (n = 10), low grade PCa (n = 10), and high grade PCa (n = 10) (33). Men were classified as having low versus high grade PCa by GS, where GS = 6 was considered low grade and GS = 8-10 was considered high grade. The most common glycan species detected in all samples was the biantennary complex glycan, NeuAc2Gal2N2M3N2, with and without core fucose, which is the most common glycan species found on PSA (**Figure 2**) (33). A global decrease in tri- and tetra-antennary glycans and an increase in bisecting *N*-acetylglucosamine was found to be correlated with disease severity. Interestingly, structures with bisecting *N*-acetylglucosamine prevent branching and therefore, metastasis, so their increased presence in EPS derived from high grade PCa is counterintuitive to what is currently reported in the literature (33).

As all these studies were performed on different sources (tissue, serum, EPS, EPS-urine), it is difficult to make definitive conclusions at the present time as to how *N*-glycosylation changes as PCa progresses. However, general trends observed from these collective studies suggest an increase in complex and high mannose *N*-glycans in PCa relative to benign prostate. In addition, increased branching may occur with disease progression and may be associated with CRPC. Furthermore, increased α2-3 sialylation as well as core fucosylation appear to be highly associated with PCa and the sialyl lewis X and lewis Y antigens appear correlated with metastatic disease.

### *N*-Glycosylation Changes of Common, PCa Markers

#### I. PSA

The most common screening test for PCa is serum PSA where levels > 4 ng/mL are considered to be abnormal (14). Although serum concentrations higher than 10 ng/mL are highly specific for PCa, most men with abnormal results are found to have only mild elevations on initial screening (i.e. 4-10 ng/mL) where only ~25% of men will be confirmed to have cancer on biopsy (14, 15). Furthermore, PSA levels alone cannot distinguish which tumors are favored to remain indolent versus progress to metastasis, often resulting in unnecessary treatment for men with cancer that would otherwise not affect quality of life or shorten life span (14, 15). Despite its limitations, PSA remains the most commonly used clinical test for PCa screening and monitoring treatment response (14). Importantly, PSA has a confirmed single site for *N*-glycosylation (Asn-69) and changes in this glycan structure is strongly associated with and quite specific for PCa (34).

Earlier studies have characterized the major *N*-glycan on PSA as a biantennary, disialylated structure of the *N*-acetylglucosamine type (**Figure 2**) (35). A subsequent study by a different group analyzed two isoforms of PSA derived from the seminal plasma of healthy donors (PSA-A and PSA-B) and found that both isoforms had mono- and biantennary *N*-glycans and found a prevalence of 3 outer chain moieties (Galβ1-4GlcNAcβ1-, GlcNAcβ1-, GalNAcβ1-4GlcNAcβ1-) (36). Interestingly, the GalNAcβ1-4GlcNAcβ1- linkage is only found on a limited number of glycoproteins and has been proposed by other authors to potentially have an immunosuppressive effect (37, 38). Another earlier study profiled the *N*-glycan signature on PSA present in the LNCaP cell line, which is derived from a patient with metastatic PCa without prior hormonal therapy (39). In this setting, triantennary structures were present and there was an observed overall decreased in sialic acid content and increase in fucosylation and *N*-acetylglucosamine *(* 39). As PSA can be present in its free form (free-PSA) or complexed to alpha-1-antichymotrypsin (complexed PSA) (40), researchers have attempted to compare the *N*-glycans in these different molecular contexts in PCa (41, 42). Results of these studies showed no significant difference in the glycosylation profiles between free and complexed PSA and there was a high prevalence of fucosylated biantennary structures. High levels of sialylation was observed in the samples with a significant fraction found to be α2-3 linked. Although informative, most of these older studies were done on very small patient cohorts.

In 2009, White et al. utilized thiophilic absorption chromatography to enrich for PSA and prostate acid phosphatase (PAP) in seminal plasma followed by purification of each protein by SDS-PAGE (43). The seminal plasma used was derived from men with no disease (n = 65), BPH (n = 59), and PCa (n = 92). Following analysis by HPLC and MALDI-TOF, 40 glycoforms of PSA were discovered (21 for PAP) and these structures ranged from complex bi- and tetraantennary structures to hybrid and high mannose forms (43). Given the degree of variability, the authors were unable to determine disease-specific patterns and suggested that the use of pooled samples for analysis was a limiting factor in the experimental design (43). In 2016, Llop et al. utilized lectin-based assays to analyze the core fucosylation and sialylation of *N*-glycans on serum-derived PSA from men with BPH (n = 29) and PCa (n = 44) (44). Here, a significant increase in core fucose and α2-3 sialic acid PSA was found in patients with PCa. Furthermore, a cut-off value of 0.86 of the PSA core fucose ratio could distinguish between high-risk PCa (GS ≥ 8) and BPH with 90% sensitivity and 95% specificity (44). In the case of α2-3 sialic acid percentage of PSA, the cut off value of 30% distinguished between high risk PCa and the group of BPH, low risk PCa (GS ≤ 6, tPSA < 10 ng/mL, clinical stage ≤ pT2a), and intermediate risk PCa (GS = 6 with tPSA ≥ 10 ng/mL or clinical stage ≥ pT2a or GS = 7) with a sensitivity of 85.7% and specificity of 95.5% (44). The utility of α2-3 sialic acid PSA in discriminating high-risk PCa from benign or low-risk disease has been confirmed in subsequent studies and shows significant promise as a parameter for stratifying which patients require treatment and which are favored to have an indolent course (45, 46).

#### II. PAP

PAP is a 50 kDa glycoprotein which was one of the earliest biomarkers used for PCa screening before being replaced by the PSA test. In 1997, it was declared no longer clinically useful due to its lower sensitivity for detecting cancer (47%) compared to PSA (96%) (47). However, as PAP has three *N*-glycosylation sites (Asn-62, Asn-301, and Asn-188) (43, 48), there was significant interest in determining whether certain structural changes to glycans could improve sensitivity and specificity for PCa.

Asn-62 and Asn-301 have been historically characterized by X-ray crystallography as having primarily high mannose structures while Asn-188 had complex structures (48). An earlier study utilized lectin affinity chromatography to compare the *N*-glycans on PAP purified from homogenized human PCa tissue (n = 5) and BPH (n = 5) (49). Here, it was found that PCa-derived PAP had decreased high mannose structures compared to BPH with an increase in nonfucosylated hybrid structures (49). In the previously mentioned cohort studied by White et al. (43) utilizing thiophilic absorption chromatography for PSA and PAP enrichment, it was found that PAP contained 21 glycoforms. This study confirmed the presence of high mannose structures at Asn-301; however, Asn-62 was defined as having mostly sialylated complex bi and tri-antennary structures (43). Asn-188 was less characterized in this study but was thought to have a tetra-antennary structure with sialic acid and fucose content (43). A study in 2013 which analyzed expressed prostatic secretions representing different stages of prostate cancer further confirmed the presence of high mannose structures at Asn-301 with bi and tri-antennary structures present at Asn-62 and Asn-188 (33). The authors further suggested that increased bisecting *N*-acetylglucosamine, decreased branching, and the presence of Neu5GC on PAP glycans may be correlated with disease severity (33).

The cumulative studies that have examined the *N*-glycosylation status of PAP suggests that a combination of high mannose, bi, and tri-antennary structures are present between the 3 sites. Decreased branching and increased bisecting *N*-acetylglucosamine of these glycans may be associated with disease progression. Furthermore, the presence of Neu5GC on Asn-301 is significant as this sialic acid is only synthesized by non-humans due to irreversible mutation of the *CMAH* gene (50, 51). Therefore, if present on human cells, it is assumed to be obtained through dietary means and is considered immunogenic (50, 51). As PAP is highest in prostate tissue (52), this finding is deserving of further study to determine whether Neu5GC may be a target in PCa.

#### III. PSMA

Prostate-specific membrane antigen (PSMA) is a type II membrane glycoprotein (100-120 kDa) that is expressed highly in PCa and correlates with aggressiveness (53). In recent years, PSMA has received significant attention due to its important role in PCa imaging and therapy (53, 54). Despite its name, the protein is also expressed in other cell types where its enzymatic functions as a folate hydrolase and NAALADase have been widely characterized (55). In PCa, its exact function remains unclear but it is thought to be negatively regulated by androgens and has become a useful marker for hormone-refractory carcinomas and for the detection of metastases (56). Studies show that PSMA has 10 *N*-glycosylation sites, three of which are in the catalytic domain (Asn 336, Asn 459, and Asn 476) and it is predicted that 20-25% of the total molecular weight is due to carbohydrates (56). Despite having a high level of *N*-glycosylation, there are surprisingly very few studies have attempted to elucidate the specific glycan structures present on PSMA and how they are altered with disease progression. An early study used a series of exo and endoglycosidases, which target different glycan moieties, to determine what types of structures are present in LNCaP cells, patient tumor tissue lysate, and serum (57). Here, complex type glycans lacking polylactosamine were observed on PSMA derived from tissue and serum while primarily high mannose forms were observed in the LNCaP setting (57). The authors stated this discrepancy is likely due to a defect in one of the *N*-glycan biosynthetic steps where LNCaP cells are unable to convert the high mannose forms to the complex type that is observed *in vivo* (57).

Barinka et al. studied the enzymatic activity of PSMA as a consequence of glycosylation by mutating different *N*-glycosylation sites on the protein (58). The results of this study showed that mutations at Asn-76 (N76A), Asn-336 (N336A), and Asn-459 (N459A) caused an increase in activity exceeding 50% of the wild-type (58). Interestingly, Asn-336 and Asn-459 are within the catalytic domain and it was hypothesized these mutations would cause the opposite effect. For mutations at Asn-121 (N121A), Asn-140 (N140A), Asn-153 (N153A), Asn-195 (N195A), and Asn-638 (N638A), the exopeptidase activity was markedly compromised (58). In particular, N121A, N195A, and N638A caused nearly complete inactivity (58). Collectively, these studies show that PSMA is highly glycosylated and that *N*-glycosylation, particularly at distal sites, is critical to the function of the protein. Further studies are needed to characterize the specific glycan structures and what changes occur as disease progresses.

### Advances in Studying *N*-Glycopathology

Historical studies that have characterized glycosylation changes associated with disease primarily relied on chemical reactions with monosaccharide constituents, metabolic labeling of glycoconjugates with radioactive sugars to help elucidate glycan composition, as well as lectin-based assays to characterize specific structural features of glycans [for a complete review of tools used for characterizing glycans, refer to Chapter 50 of (13)]. Although these methods have proven quite useful, most of these studies are unable to characterize the entire glycan species present which has caused slow progress in defining changes to carbohydrates that occur with disease progression. Many modern studies have utilized solid phase extraction techniques/chromatography to isolate N-glycans from total protein lysates and this has allowed specific glycans to be determined in various tissue homogenates. However, as many glycans are secreted into the extracellular matrix or stroma, spatial information is lost and whether or not a specific glycan is located within the tumor or stroma becomes a significant limiting factor in the interpretation of the data. The Drake lab, in collaboration with Anand Mehta, has developed an Imaging mass spectrometry (MALDI-IMS) technique for profiling N-glycans directly on formalin-fixed, paraffin-embedded (FFPE) tissue, which has been further applied to many disease settings including PCa (18, 59). The approach involves spraying a molecular coating of PNGase-F directly on the tissue to release the N-glycans. The reaction takes place in a humidified chamber so that there is little diffusion. Following the application of enzyme, chemical matrix is applied and glycans are analyzed by MALDI at each discrete tissue location in a rastered-grid format. Glycan abundances at each tissue location is then determined and visualized using intensity maps (similar to heat maps). This powerful technology adds significant advance to the fields of glycobiology and molecular histology and since it can be applied to formats such as tissue microarray, has the ability to profile the glycans across large patient cohorts.

## Mucin-Type *O*-Linked Glycosylation and Prostate Cancer Progression

Mucin-Type *O*-linked glycosylation is a post-translational process occurring in the Golgi apparatus where a GalNAc is added to the Ser/Thr residue of a protein followed by the step-wise addition of individual monosaccharides (13). The glycans resulting from this process, which may contain any of 8 core structures (**Figure 1**), are found on cell-surface and secreted proteins and are particularly prevalent on mucins which are further described subsequently. Unlike *N*-linked glycosylation, there is no pre-formed precursor and a consensus site has yet to be determined although predictive algorithms exist (13). There have been few historical studies that have attempted to characterize the *O*-glycome in PCa due to the high number of possible *O*-glycan modifications which remains incompletely characterized (60). However, due to advances in glycoproteomics in recent years, newer studies are emerging which provide insight into how mucin-type *O*-glycosylation is altered with PCa progression.

### Broad Changes to Mucin-Type *O*-Glycosylation in PCa Progression

Studies attempting to characterize global changes to the mucin-type *O*-glycome as a function of PCa disease severity are currently very limited. In 2014, Chen et al. demonstrated that PCa has elevated levels of GCNT1, an enzyme catalyzing the formation of core-2 *O*-glycans (61). It was further shown that its increased expression was associated with increased levels of core-2 O-linked sialyl lewis X structures on PSA, MUC1, and PAP (61). Although the pathological significance of this structure on these proteins remains unknown, its presence was able to differentiate PCa from benign tissue with improved specificity compared to protein level alone (61). Interestingly, a subsequent study demonstrated that GCNT1-positive tumors were highly associated with extracapsular extension and that its detection in urine post digital rectal examination was an independent risk factor for biochemical recurrence (62). Future work is needed to determine the full utility of GCNT1 as a screening tool in predicting which tumors are favored to remain indolent versus metastasize as well as mechanisms as to how GCNT1 and its product contribute to PCa progression.

A recent study utilized surgically removed PCa tissue representing different histological grades (Grade 1-5, n = 10 cases per grade) as well as tissue from patients diagnosed with BPH (n = 5) (63). Here, 17 structures covering 13 compositions were observed and Core-1 and -2 structures were predominant across all samples (63). A significant reduction in sialylated core-1 and an increase in sialylated core-2 structures were observed as PCa progresses. Correlation analysis between the *O*-glycome and *O*-glycoproteome further revealed that the sialylated core-2 structure found to be elevated as PCa progressed was highly correlated with collagen IV and the glycoform was confidently identified at T1627 across all 54 replicates (63). Interestingly, Core-2 structures have been linked to evasion of natural killer (NK) cell immunity in the context of PCa which is deserving of further study (64).

Truncated *O*-glycans, such as Tn or sTn antigen, are commonly found in many tumor settings and linked to poor prognosis (10, 65–67). Furthermore, these carbohydrates are immunoreactive and several agents have been developed for therapeutic targeting (65). In PCa, there is conflicting data regarding the prevalence of these antigens. However, it is has been reported that 4-26% of adenocarcinomas are positive for the Tn antigen and therefore, may qualify for Tn-targeted therapy (68). Interestingly, studies have shown that ST6GalNAC1, the enzyme that catalyzes the formation of sTn, is a direct and rapidly activated target gene of AR and transforms the cells to a more mesenchymal phenotype (69). Further studies are ultimately needed to determine the true prevalence of truncated *O*-glycans in PCa of different stages as well as the functional significance of their presence.

### Mucins

Mucins are cell-surface or secreted glycoproteins containing clusters of *O*-glycans (13). They can be antiadhesive and repel cell-surface interactions or promote adhesion by recognizing glycan binding proteins *via* their *O*-GalNAc glycans (13). Furthermore, they are known to have multiple effects on the immune system and maintain cellular homeostasis through various signaling mechanisms (13). In 2005, Cozzi et al. characterized the expression of MUC1, MUC2, MUC4, MUC5AC, and MUC6 using tissue microarray (TMA) from 120 paraffin-embedded specimens derived from patients who underwent radical prostatectomy or transurethral resection of the prostate (TURP) (70). The cases included both non-metastatic primary PCa as well as 10 matched lymph node metastases. Here, MUC1 overexpression was found in 58% of primary PCa and 90% of lymph node metastases but not in normal adult or benign tissues (70). In addition, 86% of MUC1-positive tumors had GS scores > 7 (70). In another study, 57 biopsy specimens from PCa patients treated with hormone therapy as well as 10 normal cases were collected and stained for sialyl-Tn MUC-1 (71). Here, it was found that the level of sialyl-Tn MUC1 significantly correlated with progression-free and cause-specific survival and may predict prognosis for patients undergoing hormonal therapy (71).

Recently, Yasumizu et al. has shown that up-regulation of MUC1-C in hormone-sensitive PCa cells suppresses AR and induces neuroendocrine (NE) differentiation through up-regulation of the neural BRN2 transcription factor and suppression of the p53 pathway (72). Furthermore, another study has shown that MUC1-C directly binds to E2F1 resulting in activation of the BAF pathway and increased cancer stem cell (CSC) renewal in NE prostate cancer (NEPC) (73). These results only highlight the important role that *O*-glycans have in disease progression and the critical need for therapeutic targeting.

## *O*-GlcNAcylation

The *O*-GlcNAc modification occurs in the nuclear and cytoplasmic compartments of the cell and does not elongate to form complex structures such as what is observed in other forms of glycosylation (13). Elevated levels of UDP-GlcNAc, derived from the hexosamine biosynthesis pathway (HBP), drive *O*-GlcNAcylation through activation of the enzyme, OGT (13, 74). Similar to phosphorylation, the residue is attached and removed several times in the lifetime of a polypeptide and the process is known to have significant effects on cellular processes such as transcription, signaling, and epigenetics (13, 75).

In 2014, Gu et al. performed immunostaining for *O*-GlcNAc on several PCa tissues (n = 55) as well as adjacent benign (n = 10) and BPH (n = 19) (76). Here, it was observed that *O*-GlcNAcylation was significantly increased in PCa tissue relative to benign disease (76). Furthermore, levels of *O*-GlcNAc are found to positively correlate with GS and be associated with reduced patient survival (77). Levels of OGT have been similarly found to be overexpressed in PCa and correlated with GS (78). Furthermore, its activity has been shown to be critical to c-MYC stability and MYC-driven proliferation of PCa cells (79). In addition to the effects on c-MYC, inhibition of OGT has been linked to decreased glucose consumption, decreased lactate secretion, and has been shown to lead to suppression of CDK1, whose expression predicts PCa recurrence (80). Effectively targeting aberrant *O-*GlcNAcylation through OGT inhibition may represent a good therapeutic strategy for targeting PCa cells. However, as *O*-GlcNAcylation is a critical process to every cell type, selectivity remains a limiting factor.

## Galectins

Galectins are among the most widely expressed lectins in all organisms, typically recognizing β-galactose containing glycoconjugates (13). They have been reported to have a wide variety of biological functions including regulation of immune response, microbial recognition, as well as roles in development (13). Importantly, they are known to have roles in cancer progression and metastasis, likely through modulation of interactions between tumor cells and the surrounding microenvironment (endothelial cells, stromal cells, and immune cells) (81). In PCa, galectin-1 (Gal-1) was found to be the most abundant galectin expressed in PCa tissue with marked up-regulation as the disease progresses to CRPC (82). Interestingly, all other galectins were found to be expressed at lower levels with Gal-3, Gal-4, Gal-9, and Gal-12 becoming downregulated with disease evolution and Gal-8 remaining unchanged (82). Furthermore, Gal-1 has been shown to be highly associated with angiogenesis and use of an allosteric inhibitor (LLS30) resulted in significant growth inhibitory effects in human CRPC xenograft models (83).

In a study that examined galectin-3 (Gal-3) expression by tissue microarray constructed from 83 patients who underwent prostatectomy (83 tumor, 78 adjacent benign, and 75 benign tissues), it was shown that Gal-3 expression was significantly decreased in tumor tissue compared to benign (84). However, despite decreased expression, Gal-3 staining in tumor specimens was able to predict biochemical recurrence (PSA ≥ 0.2 ng/mL) with 91.3% sensitivity and 75% specificity, potentially implicating it as a prognostic marker (84). In 2018, Gao et al. determined that cleaved Gal-3, rather than intact Gal-3 (detected by older studies), is present in PCa tissue but completely absent in benign (85). Furthermore, cleaved Gal-3 was positively associated with tumor progression and metastasis and its expression was closely related to PSA level (85). In addition, previous studies have shown that cleaved Gal-3 was crucial to bone remodeling in the metastatic niche implicating it as a potential therapeutic target for men with metastatic disease (86). Future studies are needed to further determine the pathological significance of cleaved Gal-3 and whether therapeutic targeting could be beneficial.

The role of other galectins in PCa progression remains controversial. Although Gal-4 was reported previously to have decreased expression with PCa progression (82), some authors have found that expression of both Gal-4 and C1GALT1 together predicts poor overall survival (87). Furthermore, Gal-4 was found to interact with C1GALT1-dependent *O*-glycans resulting in castration resistance through activation of receptor tyrosine kinase signaling and SOX9 (88). Gal-8, which remains stably expressed throughout PCa development and progression, was shown to contribute to metastasis through rearrangement of the cytoskeleton and modulation of E-Cadherin expression (89). Future studies are ultimately needed to determine the therapeutic benefit of targeting galectins in the context of PCa in relation to effects on patient survival as well as the toxicity of such an approach.

## Glycoconjugates

Glycoconjugates are carbohydrates that are covalently linked to other biological molecules, such as proteins (glycoproteins) and lipids (glycolipids) (13). These molecules make up the majority of the cell surface (termed the “glycocalyx”) and have significant effects on regulating cell-cell interactions, interactions with the extracellular matrix, as well as intracellular signaling to control a wide-variety of cellular functions (13). In this section, major studies are summarized that highlight the functional consequences of glycoconjugate expression in the context of PCa, both as potential tumor drivers and also tumor suppressors.

### Proteoglycans

Proteoglycans are heavily glycosylated proteins, consisting of both a core protein and one or more glycosaminoglycan (GAG) chains, such as heparan sulfate, chondroitin sulfate, or dermatan sulfate (13). They can be located on the cell-surface or secreted into the extracellular matrix and have significant contributions to intracellular signaling and cell-cell interactions (13). Due to these functions, they have a high degree of control over proliferation and apoptosis and have been implicated in many tumor settings including PCa. For example, an earlier study showed that the CSPG, versican, was superior to tumor grade in predicting progression in patients with early stage PCa (90). Furthermore, the anti-adhesive properties of versican were observed to cause PCa cells to lose attachment to fibronectin, a major component of the stroma, which can increase the metastatic potential of cells (91). Subsequent studies have shown that versican contains AR-response elements and is positively regulated by AR, suggesting this may be a critical downstream mediator of AR-driven signaling (92). Biglycan, a proteoglycan of the extracellular matrix, was found to be expressed in 78% of 11,070 PCa tumors and was linked to the presence of TMPRSS2:ERG fusion and PTEN deletion (93). In addition, although not functionally proven, its expression was strongly linked to AR levels suggesting androgen regulation (93). Perlecan, a basement-membrane specific HSPG, has been shown to be up-regulated in PCa, positively correlating with Gleason score and Ki67 indices (94). Furthermore, its effects have been shown to be due to its ability to regulate sonic hedgehog (HH) signaling, which was found to occur independently of androgen (94). Other studies have shown that the HSPG, Syndecan-1, is an independent predictor of poor survival (95, 96), associated with the epithelial to mesenchymal transmission (97), and may be associated with resistance to docetaxel chemotherapy (98).

In addition to promoting tumor growth in certain settings, several proteoglycans have been reported as tumor suppressors. Glypican-5, a member of the HSPG family, was shown to be lowly expressed in PCa cell lines and its overexpression significantly inhibited cell proliferation and invasion through inhibition of EMT and Wnt/β-catenin signaling (99). Glypican-1 was shown by Quach et al. to suppress proliferation in DU-145 cells but promote it in PC-3 cells (100). Interestingly, when PC-3 was either grown *in vivo* or co-cultured with stromal cells, Glypican-1 was found to prevent tumor growth suggesting a cell-dependent role as well as highlighting the importance of the tumor environment in affecting the activity of proteoglycans (100). In addition to glypicans 1 and 5, decorin has been observed to be negatively associated with PCa (90, 101) and was shown to suppress tumor growth through inhibition of EGFR and AR phosphorylation, leading to inhibition of PI3K/AKT, a critical pathway in PCa (102). Lumican, a small leucine-rich proteoglycan of the extracellular matrix, has also been shown to inhibit PCa progression when present in the reactive stroma, potentially implicating it as a positive prognostic marker (103). Overall, the roles that various proteoglycans have on PCa progression is extremely complex and is significantly influenced by the tumor microenvironment. A combination of both *in vitro* and high quality *in vivo* models are needed to fully understand the role specific proteoglycans have in the evolution of PCa.

### Glycosphingolipids

Glycosphingolipids (GSLs) are the major class of glycolipids found in animals (13). They consist of a hydrophobic ceramide backbone linked to a hydrophilic carbohydrate moiety and are responsible for maintaining the stability of cell membranes as well as regulating numerous cellular processes (proliferation, apoptosis, cell-cell-adhesion, migration, etc) (13, 104). An earlier study extracted the glycolipids from human prostate tissue surgically derived from six patients with BPH (105). Here, abundant neutral mono- to tetraglycosylceramides were obtained in addition to mono and disialylgangliosides (105). In a 2004 study, three AR-negative cell lines (PC-3, DU-145, and HH870) and two AR-positive cell lines (LNCaP-FGC, LNCaP-FGC 10) were used to characterize the gangliosides on both 2-D chromatograms as well as by confocal fluorescent microscopy (106). It was found that AR-negative cells expressed higher levels of GM1b and GD1a relative to AR-positive cells. Furthermore, O-AcGD2 was specific to the AR-negative cell lines, PC-3 and HH870 (106). A follow-up study profiled the IgM responses to 8 gangliosides (GM3, GM2, GD3, GD2, GD1a, GM1a, GD1b, GT1b) in the sera of patients with BPH (n = 11), organ-confined PCa (T1/T2, n = 36), PCa with extra-prostatic extension (T3/T4, n = 27), and age-matched healthy controls (n = 11) (107). Patients with PCa showed increased titers against GD1a and decreased titers against GD3 (107). Interestingly, patients with organ-confined PCa showed increased titers against GD1a relative to unconfined PCa despite the previous study showing cell lines with more advanced phenotype (PC-3, DU-145, and HH870) showing elevated levels of the ganglioside. The authors conclude this may be due to B cells recognizing tumor-derived GD1a as a “danger signal”, producing IgM to target GD1a-positive cells, contributing to the decline in GD1a as disease progresses (107). The specific role of GD1a in PCa remains unknown; however, its expression has been suggested to be controlled indirectly by NF-κB through transcriptional regulation of ST3GalI, II, and III, which are critical to its biosynthesis (108).

## Conclusions and Perspectives

**Figure 3:**
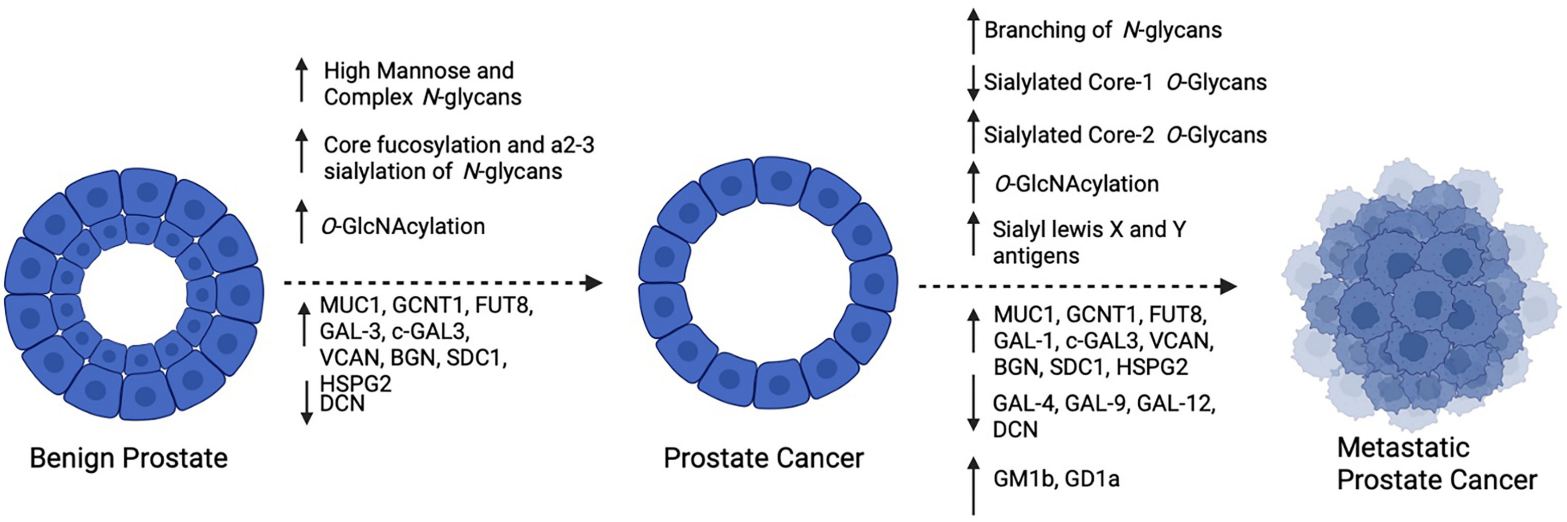
Summary of reported changes in glycosylation as PCa progresses: As PCa develops, high mannose and complex biantennary *N*-glycans become prevalent, with frequent core fucosylation and a2-3 sialylation of complex forms, Furthermore, increased *O*-GlcNAcylation and altered expression of glycosyltransferases, glycoconjugates and galectins occurs. As PCa becomes metastatic, increased branching of *N*-Glycans and alterations in *O*-Glycans occurs with frequent expression of sialyl lewis X and Y antigens. In addition to changes in glycosyltransferases, glycoconjugates and galectin expression, glycolipids GM1b and GD1a have been reported to be increased. Created with BioRender.com.

It is well accepted that aberrant glycosylation is a hallmark of cancer and has a significant effect on tumor progression. As the prostate is a major producer of glycoproteins, it is not surprising that several structural changes to glycans and changes in the expression of glycoconjugates are observed throughout the evolution of PCa (**Figure 3**) and that these appear to be critical to disease progression. Furthermore, as shown by Munkley et al. (109), several enzymes involved in the synthesis and degradation of glycans are directly regulated by androgen stimulation, suggesting that downstream glycosylation is very likely an important mediator of PCa activity deserving of further study. Due to technological limitations, as well as small sample sizes used for glycan profiling in many of these studies, progress in defining changes to the PCa tumor glycome has been slow. There are many unanswered questions in the field of PCa including how to predict which patients will have indolent versus aggressive disease, why hormonally-treated tumors become resistant to therapy, how NE differentiation occurs, and why PCa tumors can evade immunosurveillance. Despite a surge in RNA-sequencing and proteomic studies that have included large cohorts of men with PCa in different disease stages, these questions remain unanswered and many of the molecular drivers that are discovered in these studies are “un-druggable”. Therefore, there is a need to survey other molecular changes that occur as PCa progresses in addition to the products of gene expression. With greater realization of the contribution of carbohydrates to disease, the development of glycan-targeted therapy has become a high area of interest in the pharmaceutical community (110). These agents includes antibodies, enzyme inhibitors, as well as compounds that can disrupt glycan-protein interactions. In the field of PCa, it is of critical importance to thoroughly define the PCa glycome in different disease stages so that as these agents become widely available, they can be applied to patients with advanced PCa. A deeper understanding as to how glycosylation changes as PCa progresses may shed light on some of the major unanswered questions in the field and provide more opportunities for therapeutic intervention ultimately improving patient survival.

## Author Contributions

WB wrote the manuscript. JH supervised WB and edited the manuscript. All authors contributed to the article and approved the submitted version.

## Conflict of Interest

The authors declare that the research was conducted in the absence of any commercial or financial relationships that could be construed as a potential conflict of interest.

The handling editor declared a past co-authorship with one of the authors JH.

## Publisher’s Note

All claims expressed in this article are solely those of the authors and do not necessarily represent those of their affiliated organizations, or those of the publisher, the editors and the reviewers. Any product that may be evaluated in this article, or claim that may be made by its manufacturer, is not guaranteed or endorsed by the publisher.

## References

[1] Miller KD Nogueira L Mariotto AB Rowland JH Yabroff KR Alfano CM. Cancer Treatment and Survivorship Statistics, *2019* . CA Cancer J Clin (2019) 69(5):363–85. doi: 10.3322/caac.21565 31184787

[2] Hussain A Dawson N. Management of Advanced/Metastatic Prostate Cancer: 2000 Update. Oncol (Williston Park) (2000) 14(12):1677–88; discussion 1688, 1691-4. 11204373

[3] Barry MJ Nelson JB. Patients Present With More Advanced Prostate Cancer Since the USPSTF Screening Recommendations. J Urol (2015) 194(6):1534–6. doi: 10.1016/j.juro.2015.09.033 26384450

[4] Culig Z Santer FR. Androgen Receptor Signaling in Prostate Cancer. Cancer Metastasis Rev (2014) 33(2-3):413–27. doi: 10.1007/s10555-013-9474-0 24384911

[5] Teo MY Rathkopf DE Kantoff P. Treatment of Advanced Prostate Cancer. Annu Rev Med (2019) 70:479–99. doi: 10.1146/annurev-med-051517-011947 PMC6441973 30691365

[6] Sinha A Huang V Livingstone J Wang J Fox NS Kurganovs N. The Proteogenomic Landscape of Curable Prostate Cancer. Cancer Cell (2019) 35(3):414–427 e6. doi: 10.1016/j.ccell.2019.02.005 30889379 PMC6511374

[7] Pritchard CC Mateo J Walsh MF De Sarkar N Abida W Beltran H. Inherited DNA-Repair Gene Mutations in Men With Metastatic Prostate Cancer. N Engl J Med (2016) 375(5):443–53. doi: 10.1056/NEJMoa1603144 PMC4986616 27433846

[8] Robinson D Van Allen EM Wu YM Schultz N Lonigro RJ Mosquera JM. Integrative Clinical Genomics of Advanced Prostate Cancer. Cell (2015) 161(5):1215–28. doi: 10.1016/j.cell.2015.05.001 PMC4484602 26000489

[9] Chen S Zhu G Yang Y Wang F Xiao YT Zhang N. Single-Cell Analysis Reveals Transcriptomic Remodellings in Distinct Cell Types That Contribute to Human Prostate Cancer Progression. Nat Cell Biol (2021) 23(1):87–98. doi: 10.1038/s41556-020-00613-6 33420488

[10] Pinho SS Reis CA. Glycosylation in Cancer: Mechanisms and Clinical Implications. Nat Rev Cancer (2015) 15(9):540–55. doi: 10.1038/nrc3982 26289314

[11] Drake RR Jones EE Powers TW Nyalwidhe JO. Altered Glycosylation in Prostate Cancer. Adv Cancer Res (2015) 126:345–82. doi: 10.1016/bs.acr.2014.12.001 25727153

[12] Mehta AY Cummings RD. GlycoGlyph: A Glycan Visualizing, Drawing and Naming Application. Bioinformatics (2020) 36(11):3613–4. 10.1093/bioinformatics/btaa190 PMC7267839 32170934

[13] Varki A Cummings RD Esko J Stanley P Hart GW Aebi M., editors In Essentials of Glycobiology. Cold Spring Harbor, NY: Cold Spring Harbor Laboratory Press (2015). 27010055

[14] Schatten H. Brief Overview of Prostate Cancer Statistics, Grading, Diagnosis and Treatment Strategies. Adv Exp Med Biol (2018) 1095:1–14. doi: 10.1007/978-3-319-95693-0_1 30229546

[15] Sehn JK. Prostate Cancer Pathology: Recent Updates and Controversies. Mo Med (2018) 115(2):151–5. PMC6139855 30228708

[16] Chen J Xi J Tian Y Bova GS Zhang H. Identification, Prioritization, and Evaluation of Glycoproteins for Aggressive Prostate Cancer Using Quantitative Glycoproteomics and Antibody-Based Assays on Tissue Specimens. Proteomics (2013) 13(15):2268–77. doi: 10.1002/pmic.201200541 PMC4235679 23716368

[17] Liu Y Chen J Sethi A Li QK Chen L Collins B. Glycoproteomic Analysis of Prostate Cancer Tissues by SWATH Mass Spectrometry Discovers N-Acylethanolamine Acid Amidase and Protein Tyrosine Kinase 7 as Signatures for Tumor Aggressiveness. Mol Cell Proteomics (2014) 13(7):1753–68. doi: 10.1074/mcp.M114.038273 PMC4083113 24741114

[18] Powers TW Neely BA Shao Y Tang H Troyer DA Mehta AS. MALDI Imaging Mass Spectrometry Profiling of N-Glycans in Formalin-Fixed Paraffin Embedded Clinical Tissue Blocks and Tissue Microarrays. PloS One (2014) 9(9):e106255. doi: 10.1371/journal.pone.0106255 25184632 PMC4153616

[19] Conroy LR Stanback AE Young LEA Clarke HA Austin GL Liu J. In Situ Analysis of N-Linked Glycans as Potential Biomarkers of Clinical Course in Human Prostate Cancer. Mol Cancer Res (2021) 19(10):1727–38. doi: 10.1158/1541-7786.MCR-20-0967 PMC8492534 34131069

[20] Jørgensen T Berner A Kaalhus O Tveter KJ Danielsen HE Bryne M. Up-Regulation of the Oligosaccharide Sialyl LewisX: A New Prognostic Parameter in Metastatic Prostate Cancer. Cancer Res (1995) 55(9):1817–9. 7728744

[21] Culig Z Hittmair A Hobisch A Bartsch G Klocker H Pai LH. Expression of Lewis Carbohydrate Antigens in Metastatic Lesions From Human Prostatic Carcinoma. Prostate (1998) 36(3):162–7. doi: 10.1002/(SICI)1097-0045(19980801)36:3<162::AID-PROS3>3.0.CO;2-J 9687987

[22] Martensson S Bigler SA Brown M Lange PH Brawer MK Hakomori S. Sialyl-Lewis(x) and Related Carbohydrate Antigens in the Prostate. Hum Pathol (1995) 26(7):735–9. doi: 10.1016/0046-8177(95)90220-1 7628844

[23] Walker PD Karnik S deKernion JB Pramberg JC. Cell Surface Blood Group Antigens in Prostatic Carcinoma. Am J Clin Pathol (1984) 81(4):503–6. doi: 10.1093/ajcp/81.4.503 6702753

[24] Young WW Jr Mills SE Lippert MC Ahmed P Lau SK. Deletion of Antigens of the Lewis a/B Blood Group Family in Human Prostatic Carcinoma. Am J Pathol (1988) 131(3):578–86. PMC1880693 2454582

[25] Saldova R Fan Y Fitzpatrick JM William R Watson G Rudd PM. Core Fucosylation and Alpha2-3 Sialylation in Serum N-Glycome Is Significantly Increased in Prostate Cancer Comparing to Benign Prostate Hyperplasia. Glycobiology (2011) 21(2):195–205. doi: 10.1093/glycob/cwq147 20861084

[26] Kyselova Z Mechref Y Al Bataineh MM Dobrolecki LE Hickey RJ Vinson J. Alterations in the Serum Glycome Due to Metastatic Prostate Cancer. J Proteome Res (2007) 6(5):1822–32. doi: 10.1021/pr060664t PMC3685170 17432893

[27] Hoti N Lih TS Pan J Zhou Y Yang G Deng A. A Comprehensive Analysis of FUT8 Overexpressing Prostate Cancer Cells Reveals the Role of EGFR in Castration Resistance. Cancers (Basel) (2020) 12(2). doi: 10.3390/cancers12020468 PMC7072180 32085441

[28] Gilgunn S Murphy K Stockmann H Conroy PJ Murphy TB Watson RW. Glycosylation in Indolent, Significant and Aggressive Prostate Cancer by Automated High-Throughput N-Glycan Profiling. Int J Mol Sci (2020) 21(23). doi: 10.3390/ijms21239233 PMC7730228 33287410

[29] Ishibashi Y Tobisawa Y Hatakeyama S Ohashi T Tanaka M Narita S. Serum Tri- and Tetra-Antennary N-Glycan Is a Potential Predictive Biomarker for Castration-Resistant Prostate Cancer. Prostate (2014) 74(15):1521–9. doi: 10.1002/pros.22869 25154914

[30] Drake RR Elschenbroich S Lopez-Perez O Kim Y Ignatchenko V Ignatchenko A. In-Depth Proteomic Analyses of Direct Expressed Prostatic Secretions. J Proteome Res (2010) 9(5):2109–16. doi: 10.1021/pr1001498 PMC2869496 20334419

[31] Kim Y Ignatchenko V Yao CQ Kalatskaya I Nyalwidhe JO Lance RS. Identification of Differentially Expressed Proteins in Direct Expressed Prostatic Secretions of Men With Organ-Confined Versus Extracapsular Prostate Cancer. Mol Cell Proteomics (2012) 11(12):1870–84. doi: 10.1074/mcp.M112.017889 PMC3518113 22986220

[32] Principe S Kim Y Fontana S Ignatchenko V Nyalwidhe JO Lance RS. Identification of Prostate-Enriched Proteins by In-Depth Proteomic Analyses of Expressed Prostatic Secretions In Urine. J Proteome Res (2012) 11(4):2386–96. doi: 10.1021/pr2011236 PMC3642074 22339264

[33] Nyalwidhe JO Betesh LR Powers TW Jones EE White KY Burch TC. Increased Bisecting N-Acetylglucosamine and Decreased Branched Chain Glycans of N-Linked Glycoproteins in Expressed Prostatic Secretions Associated With Prostate Cancer Progression. Proteomics Clin Appl (2013) 7(9-10):677–89. doi: 10.1002/prca.201200134 PMC3969874 23775902

[34] Stura EA Muller BH Bossus M Michel S Jolivet-Reynaud C Ducancel F. Crystal Structure of Human Prostate-Specific Antigen in a Sandwich Antibody Complex. J Mol Biol (2011) 414(4):530–44. doi: 10.1016/j.jmb.2011.10.007 22037582

[35] Belanger A van Halbeek H Graves HC Grandbois K Stamey TA Huang L. Molecular Mass and Carbohydrate Structure of Prostate Specific Antigen: Studies for Establishment of an International PSA Standard. Prostate (1995) 27(4):187–97. doi: 10.1002/pros.2990270403 7479385

[36] Okada T Sato Y Kobayashi N Sumida K Satomura S Matsuura S. Structural Characteristics of the N-Glycans of Two Isoforms of Prostate-Specific Antigens Purified From Human Seminal Fluid. Biochim Biophys Acta (2001) 1525(1-2):149–60. doi: 10.1016/S0304-4165(00)00182-3 11342264

[37] Dell A Morris HR Easton RL Panico M Patankar M Oehniger S. Structural Analysis of the Oligosaccharides Derived From Glycodelin, a Human Glycoprotein With Potent Immunosuppressive and Contraceptive Activities. J Biol Chem (1995) 270(41):24116–26. doi: 10.1074/jbc.270.41.24116 7592613

[38] Nimtz M Grabenhorst E Conradt HS Sanz L Calvete JJ. Structural Characterization of the Oligosaccharide Chains of Native and Crystallized Boar Seminal Plasma Spermadhesin PSP-I and PSP-II Glycoforms. Eur J Biochem (1999) 265(2):703–18. doi: 10.1046/j.1432-1327.1999.00766.x 10504403

[39] Peracaula R Tabares G Royle L Harvey DJ Dwek RA Rudd PM. Altered Glycosylation Pattern Allows the Distinction Between Prostate-Specific Antigen (PSA) From Normal and Tumor Origins. Glycobiology (2003) 13(6):457–70. doi: 10.1093/glycob/cwg041 12626390

[40] Oesterling JE Jacobsen SJ Klee GG Pettersson K Piironen T Abrahamsson PA. Free, Complexed and Total Serum Prostate Specific Antigen: The Establishment of Appropriate Reference Ranges for Their Concentrations and Ratios. J Urol (1995) 154(3):1090–5. doi: 10.1016/S0022-5347(01)66984-2 7543605

[41] Tabares G Jung K Reiche J Stephan C Lein M Peracaula R. Free PSA Forms in Prostatic Tissue and Sera of Prostate Cancer Patients: Analysis by 2-DE and Western Blotting of Immunopurified Samples. Clin Biochem (2007) 40(5-6):343–50. doi: 10.1016/j.clinbiochem.2006.12.006 17306785

[42] Tajiri M Ohyama C Wada Y. Oligosaccharide Profiles of the Prostate Specific Antigen in Free and Complexed Forms From the Prostate Cancer Patient Serum and in Seminal Plasma: A Glycopeptide Approach. Glycobiology (2008) 18(1):2–8. doi: 10.1093/glycob/cwm117 17956937

[43] White KY Rodemich L Nyalwidhe JO Comunale MA Clements MA Lance RS. Glycomic Characterization of Prostate-Specific Antigen and Prostatic Acid Phosphatase in Prostate Cancer and Benign Disease Seminal Plasma Fluids. J Proteome Res (2009) 8(2):620–30. doi: 10.1021/pr8007545 PMC2651839 19128049

[44] Llop E Ferrer-Batalle M Barrabes S Guerrero PE Ramirez M Saldova R. Improvement of Prostate Cancer Diagnosis by Detecting PSA Glycosylation-Specific Changes. Theranostics (2016) 6(8):1190–204. doi: 10.7150/thno.15226 PMC4893645 27279911

[45] Ferrer-Batalle M Llop E Ramirez M Aleixandre RN Saez M Comet J. Comparative Study of Blood-Based Biomarkers, Alpha2,3-Sialic Acid PSA and PHI, for High-Risk Prostate Cancer Detection. Int J Mol Sci (2017) 18(4). doi: 10.3390/ijms18040845 PMC5412429 28420168

[46] Ishikawa T Yoneyama T Tobisawa Y Hatakeyama S Kurosawa T Nakamura K. An Automated Micro-Total Immunoassay System for Measuring Cancer-Associated Alpha2,3-Linked Sialyl N-Glycan-Carrying Prostate-Specific Antigen May Improve the Accuracy of Prostate Cancer Diagnosis. Int J Mol Sci (2017) 18(2). doi: 10.3390/ijms18020470 PMC5344002 28241428

[47] Stamey TA Yang N Hay AR McNeal JE Freiha FS Redwine E. Prostate-Specific Antigen as a Serum Marker for Adenocarcinoma of the Prostate. N Engl J Med (1987) 317(15):909–16. doi: 10.1056/NEJM198710083171501 2442609

[48] Jakob CG Lewinski K Kuciel R Ostrowski W Lebioda L. Crystal Structure of Human Prostatic Acid Phosphatase. Prostate (2000) 42(3):211–8. doi: 10.1002/(SICI)1097-0045(20000215)42:3<211::AID-PROS7>3.0.CO;2-U 10639192

[49] Yoshida KI Honda M Arai K Hosoya Y Moriguchi H Sumi S. Serial Lectin Affinity Chromatography With Concavalin A and Wheat Germ Agglutinin Demonstrates Altered Asparagine-Linked Sugar-Chain Structures of Prostatic Acid Phosphatase in Human Prostate Carcinoma. J Chromatogr B BioMed Sci Appl (1997) 695(2):439–43. doi: 10.1016/S0378-4347(97)00186-2 9300882

[50] Altman MO Gagneux P. Absence of Neu5Gc and Presence of Anti-Neu5Gc Antibodies in Humans-An Evolutionary Perspective. Front Immunol (2019) 10:789. doi: 10.3389/fimmu.2019.00789 31134048 PMC6524697

[51] Yehuda S Padler-Karavani V. Glycosylated Biotherapeutics: Immunological Effects of N-Glycolylneuraminic Acid. Front Immunol (2020) 11:21. doi: 10.3389/fimmu.2020.00021 32038661 PMC6989436

[52] Hassan MI Aijaz A Ahmad F. Structural and Functional Analysis of Human Prostatic Acid Phosphatase. Expert Rev Anticancer Ther (2010) 10(7):1055–68. doi: 10.1586/era.10.46 20645695

[53] Bouchelouche K Choyke PL Capala J. Prostate Specific Membrane Antigen- a Target for Imaging and Therapy With Radionuclides. Discov Med (2010) 9(44):55–61. 20102687 PMC3410553

[54] Czarniecki M Mena E Lindenberg L Cacko M Harmon S Radtke JP. Keeping Up With the Prostate-Specific Membrane Antigens (PSMAs): An Introduction to a New Class of Positron Emission Tomography (PET) Imaging Agents. Transl Androl Urol (2018) 7(5):831–43. doi: 10.21037/tau.2018.08.03 PMC6212618 30456186

[55] Ghosh A Heston WD. Tumor Target Prostate Specific Membrane Antigen (PSMA) and its Regulation in Prostate Cancer. J Cell Biochem (2004) 91(3):528–39. doi: 10.1002/jcb.10661 14755683

[56] Ghosh A Wang X Klein E Heston WDW. Novel Role of Prostate-Specific Membrane Antigen in Suppressing Prostate Cancer Invasiveness. Cancer Res (2005) 65(3):727–31. 15705868

[57] Holmes EH Greene TG Tino WT Boynton AL Aldape HC Misrock SL. Analysis of Glycosylation of Prostate-Specific Membrane Antigen Derived From LNCaP Cells, Prostatic Carcinoma Tumors, and Serum From Prostate Cancer Patients. Prostate Suppl (1996) 7:25–9. doi: 10.1002/(SICI)1097-0045(1996)7+<25::AID-PROS3>3.0.CO;2-I 8950359

[58] Barinka C Sacha P Sklenar J Man P Bezouska K Slusher BS. Identification of the N-Glycosylation Sites on Glutamate Carboxypeptidase II Necessary for Proteolytic Activity. Protein Sci (2004) 13(6):1627–35. doi: 10.1110/ps.04622104 PMC2279971 15152093

[59] Powers TW Jones EE Betesh LR Romano PR Gao P Copland JA. Matrix Assisted Laser Desorption Ionization Imaging Mass Spectrometry Workflow for Spatial Profiling Analysis of N-Linked Glycan Expression in Tissues. Anal Chem (2013) 85(20):9799–806. doi: 10.1021/ac402108x PMC3969840 24050758

[60] Steentoft C Vakhrushev SY Joshi HJ Kong Y Vester-Christensen MB Schjoldager KTBG. Precision Mapping of the Human O-GalNAc Glycoproteome Through SimpleCell Technology. EMBO J (2013) 32(10):1478–88. doi: 10.1038/emboj.2013.79 PMC3655468 23584533

[61] Chen Z Gulzar ZG St Hill CA Walcheck B Brooks JD. Increased Expression of GCNT1 is Associated With Altered O-Glycosylation of PSA, PAP, and MUC1 in Human Prostate Cancers. Prostate (2014) 74(10):1059–67. doi: 10.1002/pros.22826 PMC5862140 24854630

[62] Kojima Y Yoneyama T Hatakeyama S Mikami J Sato T Mori K. Detection of Core2 Beta-1,6-N-Acetylglucosaminyltransferase in Post-Digital Rectal Examination Urine Is a Reliable Indicator for Extracapsular Extension of Prostate Cancer. PloS One (2015) 10(9):e0138520. doi: 10.1371/journal.pone.0138520 26390303 PMC4577128

[63] Kawahara R Recuero S Srougi M Leite KRM Thaysen-Andersen M Palmisano G. The Complexity and Dynamics of the Tissue Glycoproteome Associated With Prostate Cancer Progression. Mol Cell Proteomics (2021) 20:100026. doi: 10.1074/mcp.RA120.002320 33127837 PMC8010466

[64] Okamoto T Yoneyama MS Hatakeyama S Mori K Yamamoto H Koie T. Core2 O-Glycan-Expressing Prostate Cancer Cells Are Resistant to NK Cell Immunity. Mol Med Rep (2013) 7(2):359–64. doi: 10.3892/mmr.2012.1189 PMC3573034 23165940

[65] Springer GF. Immunoreactive T and Tn Epitopes in Cancer Diagnosis, Prognosis, and Immunotherapy. J Mol Med (Berl) (1997) 75(8):594–602. doi: 10.1007/s001090050144 9297627

[66] Springer GF Desai PR Banatwala I. Blood Group MN Antigens and Precursors in Normal and Malignant Human Breast Glandular Tissue. J Natl Cancer Inst (1975) 54(2):335–9. 163330

[67] Springer GF and Tn T. General Carcinoma Autoantigens. Science (1984) 224(4654):1198–206. doi: 10.1126/science.6729450 6729450

[68] Li Q Anver MR Butcher DO Gildersleeve JC. Resolving Conflicting Data on Expression of the Tn Antigen and Implications for Clinical Trials With Cancer Vaccines. Mol Cancer Ther (2009) 8(4):971–9. doi: 10.1158/1535-7163.MCT-08-0934 PMC2752371 19372570

[69] Munkley J Oltean S Vodak D Wilson BT Livermore KE Zhou Y. The Androgen Receptor Controls Expression of the Cancer-Associated sTn Antigen and Cell Adhesion Through Induction of ST6GalNAc1 in Prostate Cancer. Oncotarget (2015) 6(33):34358–74. doi: 10.18632/oncotarget.6024 PMC4741458 26452038

[70] Cozzi PJ Wang J Delprado W Perkins AC Allen BJ Russell PJ. MUC1, MUC2, MUC4, MUC5AC and MUC6 Expression in the Progression of Prostate Cancer. Clin Exp Metastasis (2005) 22(7):565–73. doi: 10.1007/s10585-005-5376-z 16475027

[71] Arai T Fujita K Fujime M Irimura T. Expression of Sialylated MUC1 in Prostate Cancer: Relationship to Clinical Stage and Prognosis. Int J Urol (2005) 12(7):654–61. doi: 10.1111/j.1442-2042.2005.01112.x 16045558

[72] Yasumizu Y Rajabi H Jin C Hata T Pitroda S Long MD. MUC1-C Regulates Lineage Plasticity Driving Progression to Neuroendocrine Prostate Cancer. Nat Commun (2020) 11(1):338. doi: 10.1038/s41467-020-14808-w 31953400 PMC6969104

[73] Hagiwara M Yasumizu Y Yamashita N Rajabi H Fushimi A Long MD. MUC1-C Activates the BAF (mSWI/SNF) Complex in Prostate Cancer Stem Cells. Cancer Res (2021) 81(4):1111–22. doi: 10.1158/0008-5472.CAN-20-2588 PMC8026569 33323379

[74] Akella NM Ciraku L Reginato MJ. Fueling the Fire: Emerging Role of the Hexosamine Biosynthetic Pathway in Cancer. BMC Biol (2019) 17(1):52. doi: 10.1186/s12915-019-0671-3 31272438 PMC6610925

[75] Aquino-Gil M Pierce A Perez-Cervera Y Zenteno E Lefebvre T. OGT: A Short Overview of an Enzyme Standing Out From Usual Glycosyltransferases. Biochem Soc Trans (2017) 45(2):365–70. doi: 10.1042/BST20160404 28408476

[76] Gu Y Gao J Han C Zhang X Liu H Ma L. O-GlcNAcylation Is Increased in Prostate Cancer Tissues and Enhances Malignancy of Prostate Cancer Cells. Mol Med Rep (2014) 10(2):897–904. doi: 10.3892/mmr.2014.2269 24865644

[77] Kamigaito T Okaneya T Kawakubo M Shimojo H Nishizawa Nakayama J. Overexpression of O-GlcNAc by Prostate Cancer Cells Is Significantly Associated With Poor Prognosis of Patients. Prostate Cancer Prostatic Dis (2014) 17(1):18–22. doi: 10.1038/pcan.2013.56 24366413

[78] Itkonen HM Minner S Guldvik IJ Sandmann MJ Tsourlakis MC Berge V. O-GlcNAc Transferase Integrates Metabolic Pathways to Regulate the Stability of C-MYC in Human Prostate Cancer Cells. Cancer Res (2013) 73(16):5277–87. doi: 10.1158/0008-5472.CAN-13-0549 23720054

[79] Itkonen HM Urbanucci A Martin SE Khan A Mathelier A Thiede B. High OGT Activity Is Essential for MYC-Driven Proliferation of Prostate Cancer Cells. Theranostics (2019) 9(8):2183–97. doi: 10.7150/thno.30834 PMC6531294 31149037

[80] Itkonen HM Gorad SS Duveau DY Martin SES Barkovskaya A Bathen TF. Inhibition of O-GlcNAc Transferase Activity Reprograms Prostate Cancer Cell Metabolism. Oncotarget (2016) 7(11):12464–76. doi: 10.18632/oncotarget.7039 PMC4914298 26824323

[81] Ebrahim AH Alalawi Z Mirandola L Rakhshanda R Dahlbeck S Nguyen D. Galectins in Cancer: Carcinogenesis, Diagnosis and Therapy. Ann Transl Med (2014) 2(9):88. doi: 10.3978/j.issn.2305-5839.2014.09.12 25405163 PMC4205868

[82] Laderach DJ Gentilini LD Giribaldi L Delgado VC Nugnes L Croci DO. A Unique Galectin Signature in Human Prostate Cancer Progression Suggests Galectin-1 as a Key Target for Treatment of Advanced Disease. Cancer Res (2013) 73(1):86–96. doi: 10.1158/0008-5472.CAN-12-1260 23108139

[83] Shih TC Liu R Wu CT Li X Xiao W Deng X. Targeting Galectin-1 Impairs Castration-Resistant Prostate Cancer Progression and Invasion. Clin Cancer Res (2018) 24(17):4319–31. doi: 10.1158/1078-0432.CCR-18-0157 PMC6125207 29666302

[84] Knapp JS Lokeshwar SD Vogel U Hennenlotter J Schwentner C Kramer MW. Galectin-3 Expression in Prostate Cancer and Benign Prostate Tissues: Correlation With Biochemical Recurrence. World J Urol (2013) 31(2):351–8. doi: 10.1007/s00345-012-0925-y 22892876

[85] Gao J Li T Mo Z Hu Y Yi Q He R. Overexpression of the Galectin-3 During Tumor Progression in Prostate Cancer and Its Clinical Implications. Int J Clin Exp Pathol (2018) 11(2):839–46. PMC6958038 31938173

[86] Nakajima K Kho DH Yanagawa T Harazono Y Hogan V Chen W. Galectin-3 Cleavage Alters Bone Remodeling: Different Outcomes in Breast and Prostate Cancer Skeletal Metastasis. Cancer Res (2016) 76(6):1391–402. doi: 10.1158/0008-5472.CAN-15-1793 PMC4863655 26837763

[87] Tzeng SF Tsai CH Chao TK Chou YC Yang YC Tsai MH. O-Glycosylation-Mediated Signaling Circuit Drives Metastatic Castration-Resistant Prostate Cancer. FASEB J (2018), fj201800687. doi: 10.1096/fj.201800687 29906246

[88] Tsai CH Tzeng SF Chao TK Tsai CY Yang YC Lee MT. Metastatic Progression of Prostate Cancer Is Mediated by Autonomous Binding of Galectin-4-O-Glycan to Cancer Cells. Cancer Res (2016) 76(19):5756–67. doi: 10.1158/0008-5472.CAN-16-0641 27485450

[89] Gentilini LD Jaworski FM Tiraboschi C Perez IG Kotler ML Chauchereau A. Stable and High Expression of Galectin-8 Tightly Controls Metastatic Progression of Prostate Cancer. Oncotarget (2017) 8(27):44654–68. doi: 10.18632/oncotarget.17963 PMC5546508 28591719

[90] Ricciardelli C Mayne K Sykes PJ Raymond WA McCaul K Marshall VR. Elevated Levels of Versican But Not Decorin Predict Disease Progression in Early-Stage Prostate Cancer. Clin Cancer Res (1998) 4(4):963–71. 9563891

[91] Sakko AJ Ricciardelli C Mayne K Suwiwat S LeBaron RG Marshall VR. Modulation of Prostate Cancer Cell Attachment to Matrix by Versican. Cancer Res (2003) 63(16):4786–91. 12941795

[92] Read JT Rahmani M Boroomand S Allahverdian S McManus BM Rennie PS. Androgen Receptor Regulation of the Versican Gene Through an Androgen Response Element in the Proximal Promoter. J Biol Chem (2007) 282(44):31954–63. doi: 10.1074/jbc.M702099200 17728259

[93] Jacobsen F Kraft J Schroeder C Hube-Magg C Kluth M Lang DS. Up-Regulation of Biglycan is Associated With Poor Prognosis and PTEN Deletion in Patients With Prostate Cancer. Neoplasia (2017) 19(9):707–15. doi: 10.1016/j.neo.2017.06.003 PMC5565634 28830008

[94] Datta MW Hernandez AM Schlicht MJ Kahler AJ DeGueme AM Dhir R. Perlecan, A Candidate Gene for the CAPB Locus, Regulates Prostate Cancer Cell Growth *via* the Sonic Hedgehog Pathway. Mol Cancer (2006) 5:9. doi: 10.1186/1476-4598-5-9 16507112 PMC1421430

[95] Sharpe B Alghezi DA Cattermole C Beresford M Bowen R Mitchard J. A Subset of High Gleason Grade Prostate Carcinomas Contain a Large Burden of Prostate Cancer Syndecan-1 Positive Stromal Cells. Prostate (2017) 77(13):1312–24. doi: 10.1002/pros.23391 28744948

[96] Szarvas T Reis H Dorp FV Tschirdewahn S Niedworok C Nyirady P. Soluble Syndecan-1 (SDC1) Serum Level as an Independent Pre-Operative Predictor of Cancer-Specific Survival in Prostate Cancer. Prostate (2016) 76(11):977–85. doi: 10.1002/pros.23186 27062540

[97] Fujii T Shimada K Tatsumi Y Tanaka N Fujimoto K Konishi N. Syndecan-1 Up-Regulates microRNA-331-3p and Mediates Epithelial-to-Mesenchymal Transition in Prostate Cancer. Mol Carcinog (2016) 55(9):1378–86. doi: 10.1002/mc.22381 26259043

[98] Szarvas T Sevcenco S Modos O Keresztes D Nyirady P Kubik A. Circulating Syndecan-1 Is Associated With Chemotherapy-Resistance in Castration-Resistant Prostate Cancer. Urol Oncol (2018) 36(6):312 e9–312 e15. doi: 10.1016/j.urolonc.2018.03.010 29628317

[99] Sun Y Xu K He M Fan G Lu H. Overexpression of Glypican 5 (GPC5) Inhibits Prostate Cancer Cell Proliferation and Invasion *via* Suppressing Sp1-Mediated EMT and Activation of Wnt/beta-Catenin Signaling. Oncol Res (2018) 26(4):565–72. doi: 10.3727/096504017X15044461944385 PMC7844840 28893348

[100] Quach ND Kaur SP Eggert MW Ingram L Ghosh D Sheth S. Paradoxical Role of Glypican-1 in Prostate Cancer Cell and Tumor Growth. Sci Rep (2019) 9(1):11478. doi: 10.1038/s41598-019-47874-2 31391540 PMC6685992

[101] Henke A Grace OC Ashley GR Stewart GD Riddick ACP Yeun H. Stromal Expression of Decorin, Semaphorin6D, SPARC, Sprouty1 and Tsukushi in Developing Prostate and Decreased Levels of Decorin in Prostate Cancer. PloS One (2012) 7(8):e42516. doi: 10.1371/journal.pone.0042516 22880013 PMC3411755

[102] Hu Y Sun H Owens RT Wu J Chen YQ Berquin IM. Decorin Suppresses Prostate Tumor Growth Through Inhibition of Epidermal Growth Factor and Androgen Receptor Pathways. Neoplasia (2009) 11(10):1042–53. doi: 10.1593/neo.09760 PMC2745670 19794963

[103] Coulson-Thomas VJ Coulson-Thomas YM Gesteira TF Andrade de Paula CA Carneiro CRW Ortiz V. Lumican Expression, Localization and Antitumor Activity in Prostate Cancer. Exp Cell Res (2013) 319(7):967–81. doi: 10.1016/j.yexcr.2013.01.023 PMC3633477 23399832

[104] Zhuo D Li X Guan F. Biological Roles of Aberrantly Expressed Glycosphingolipids and Related Enzymes in Human Cancer Development and Progression. Front Physiol (2018) 9:466. doi: 10.3389/fphys.2018.00466 29773994 PMC5943571

[105] Shiraishi T Kinter MT Mills SE Lippert MC Bova GS Young WW Jr. The Glycosphingolipids of Human Prostate Tissue. Biochim Biophys Acta (1988) 961(2):160–9. doi: 10.1016/0005-2760(88)90109-9 3390453

[106] Ravindranath MH Muthugounder S Presser N Selvan SR Portoukalian J Brosman S. Gangliosides of Organ-Confined Versus Metastatic Androgen-Receptor-Negative Prostate Cancer. Biochem Biophys Res Commun (2004) 324(1):154–65. doi: 10.1016/j.bbrc.2004.09.029 15464996

[107] Ravindranath MH Muthugounder S Presser N Ye X Brosman S Morton DL. Endogenous Immune Response to Gangliosides in Patients With Confined Prostate Cancer. Int J Cancer (2005) 116(3):368–77. doi: 10.1002/ijc.21023 15818621

[108] Hatano K Miyamoto Y Nonomura N Kaneda Y. Expression of Gangliosides, GD1a, and Sialyl Paragloboside is Regulated by NF-kappaB-Dependent Transcriptional Control of Alpha2,3-Sialyltransferase I, II, and VI in Human Castration-Resistant Prostate Cancer Cells. Int J Cancer (2011) 129(8):1838–47. doi: 10.1002/ijc.25860 21165949

[109] Munkley J Vodak D Livermore KE James K Wilson BT Knight B. Glycosylation is an Androgen-Regulated Process Essential for Prostate Cancer Cell Viability. EBioMedicine (2016) 8:103–16. doi: 10.1016/j.ebiom.2016.04.018 PMC4919605 27428423

[110] Dalziel M Crispin M Scanlan CN Zitzmann N Dwek RA. Emerging Principles for the Therapeutic Exploitation of Glycosylation. Science (2014) 343(6166):1235681. doi: 10.1126/science.1235681 24385630

